# Electrochemical Field-Effect Transistor Utilization to Study the Coupling Success Rate of Photosynthetic Protein Complexes to Cytochrome *c*

**DOI:** 10.3390/bios7020016

**Published:** 2017-03-30

**Authors:** Arash Takshi, Houman Yaghoubi, Jing Wang, Daniel Jun, J. Thomas Beatty

**Affiliations:** 1 Department of Electrical Engineering, University of South Florida (USF), Tampa, FL 33620, USA; jingw@usf.edu; 2 Department of Chemistry, University of California Irvine, Irvine, CA 92697, USA; hyaghoub@uci.edu; 3 Department of Microbiology and Immunology, University of British Columbia (UBC), Vancouver, BC V6T1Z3, Canada; djun89@gmail.com (D.J.); j.beatty@ubc.ca (J.T.B.)

**Keywords:** bio-photosensor, reaction center, rhodobacter sphaeroides, charge density, protein-protein interaction

## Abstract

Due to the high internal quantum efficiency, reaction center (RC) proteins from photosynthetic organisms have been studied in various bio-photoelectrochemical devices for solar energy harvesting. In vivo, RC and cytochrome *c* (cyt *c*; a component of the biological electron transport chain) can form a cocomplex via interprotein docking. This mechanism can be used in vitro for efficient electron transfer from an electrode to the RC in a bio-photoelectrochemical device. Hence, the success rate in coupling RCs to cyt *c* is of great importance for practical applications in the future. In this work, we use an electrochemical transistor to study the binding of the RC to cytochrome. The shift in the transistor threshold voltage was measured in the dark and under illumination to estimate the density of cytochrome and coupled RCs on the gate of the transistor. The results show that ~33% of the cyt *c*s on the transistor gate were able to effectively couple with RCs. Due to the high sensitivity of the transistor, the approach can be used to make photosensors for detecting low light intensities.

## 1. Introduction

Application of photosynthetic reaction centers (RCs) in bioelectronic devices has attracted growing interest due to the unique properties of RCs. RCs are natural protein complexes with high quantum efficiency (~100%) in generating electric charges from photons [1], which are potentially useful for making bio-photoelectrochemical devices to harvest solar energy. The conventional method of fabricating a device is to immobilize RCs on the surface of an electrode via a linker molecule and utilize the electrode in an electrochemical cell [2,3]. Recently, a new method was devised for immobilizing RCs via natural interprotein interaction between cyt *c* and RC [4,5]. Using this approach, the RC orientation is well controlled when attached to the electrode through the immobilized cyt *c* protein [4]. Additionally, using cyt *c* as a part of the linking structure can facilitate the charge transfer between the RC and the electrode [6].

**Figure 1:**
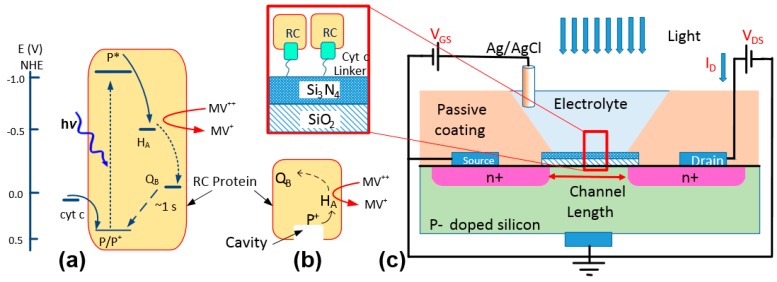
(**a**) Energy diagram demonstrating the charge circulation inside a reaction center (RC) and the interactions with cyt *c* and methyl viologen. (**b**) A schematic of an RC. The cavity indicates the cytochrome docking site. (**c**) A schematic of the electrochemical device. The device was characterized in dark and light by measuring *I_D_* while applying voltages to *V_DS_* and *V_GS_*. The structure of the immobilized proteins using a linker on the surface of Si_3_N_4_ is shown in the zoomed-in box.

The new immobilization method was previously tested on the *Rhodobacter sphaeroides* RC [4,5]. This RC is an integral membrane protein complex consisting of three subunits and cofactors. The photoreaction starts with the absorption of photons at the primary electron donor (P) which is one of the cofactors. Due to the energy profile of the cofactors, the photoexcited electron at P moves first to bacteriopheophytin (*H_A_*) and then to a quinone molecule (*Q_B_*) [1]. In a photosynthetic organism, after receiving two electrons and two protons (H^+^), *Q_B_* becomes a hydroquinone (*QH*_2_) and leaves the RC. The charge transfer from P^+^ involves cyt *c* which acts as a charge mediator [1]. Cyt *c* approaches the RC at the P-side and donates one electron to the RC. Due to the presence of Fe^2+^ ion in the cyt *c* heme, the protein has two positive charges in its reduced form. After donating an electron, cyt *c* is oxidized and carries three positive charges (Fe^3+^). One approach to remove the negative charge from the RC in an electrochemical cell is to employ a redox mediator such as methyl viologen (MV) [7]. The energy diagram in Figure 1a shows the charge balance in an electrochemical cell when an RC interacts with cyt c and MV. In this process, after absorbing a photon, the electron at P promotes from the ground state (P/P^+^) to the excited state (P*). In presence of methyl viologen the excited electron can be transferred to MV^2+^ before reaching *Q_B_*. The reduced mediator (MV^+^) can diffuse out from the protein. Meanwhile the positive charge at P^+^ will be filled with the electrons received from cyt *c*^2+^ which oxidizes the protein to cyt *c*^3+^.

The natural protein-protein interaction between the RC and cyt *c* can be used in a bio-photoelectrochemical device not only for charge transfer but also for immobilizing RCs from the P-side where the protein features a cavity (Figure 1b) [4]. Despite the advantages in this approach, one concern is the success rate in immobilizing RCs to an electrode by relying on this natural protein-protein interaction. In our previous work [4], we estimated the density of RCs on a layer of immobilized cyt *c* using a cyclic voltammetry (CV) method. However, the CV method does not reveal effective coupling between the two proteins. In this work, we have used an electrochemical field-effect transistor (FET) as a substrate to study the immobilization of RCs through cytochromes. Electrochemical FETs have been used widely as sensors for detecting various chemicals and biological materials, including proteins, DNAs, and cells [8,9,10,11,12,13]. As shown in Figure 1c, an electrochemical transistor has a structure very similar to a metal-oxide-semiconductor FET (MOSFET) but the gate metal being replaced with an electrolyte. Due to high sensitivity of the drain current, *I_D_*, to the static charge on the gate insulator, a common method is to use a linker molecule for immobilizing proteins and DNAs [10,12,13]. The immobilized materials can mimic a surface charge which can be measured accurately by monitoring the voltage shift in the device. 

## 2. Materials and Methods

Electrochemical transistors with two layers of gate insulator (98 nm thick SiO_2_ and 100 nm thick Si_3_N_4_) were purchased from Microsense. The electrochemical cells were set in disposable cuvettes with 0.1 M Tris-HCl buffer (pH 8.0) as the electrolyte and an Ag/AgCl reference electrode as the gate contact (Figure 1c).

The RC from *Rhodobacter sphaeroides* was prepared as described elsewhere [14]. The RC concentration after purification was measured to be 15 µM based on the absorption peak at 804 nm [15]. This RC has a 6-His tag on the C-terminus of the H-subunit which is on the opposite side of the RC from where cyt c binds [15]. The concentration of the RC solution was diluted to 5 µM by adding Tris-HCl buffer (pH 8.0) into the solution. A self-assembled monolayer (SAM) of the linker molecule was deposited on the Si_3_N_4_ layer of the transistor by inserting the device into an ethanolic solution of 10 mM 10-carboxydecylphosphonic acid (Dojindo, Rockville, MD, USA) for 4 h at room temperature. Cyt *c* was deposited on the SAM by inserting the device in a 0.8 mM solution of cyt *c* (Sigma, St. Louis, MO, USA) in Tris buffer (pH 8.0) for 24 h at room temperature. RC incubation was carried out by drop casting 20 μL of the diluted RC solution on top of the active area of the transistor and keeping it at 4 °C for 24 h.

The electrochemical cells were tested using a custom-made setup including a dark box with a white light emitting diode (LED) and a multi wavelength LED (MTMD6788594SMT6 from Marktech Optoelectronics, Latham, NY, USA) as the light sources inside the box. Various pins of the LEDs were biased with 20 mA current pulse to get emission for white light and single wavelength emissions at 670, 770, 810, 850, and 950 nm. From the datasheet of the LED, the intensity of the monochromatic light was estimated to be ~3 μW/cm^2^ at the transistor surface. The transistor was characterized using a 2602A Keithley instrument (Tektronix, Beaverton, OR, USA). The Keithley instrument and the pulse current source for the LEDs were controlled through LabTracer 2.0 software (Tektronix, Beaverton, OR, USA). 

## 3. Results and Discussion

**Figure 2:**
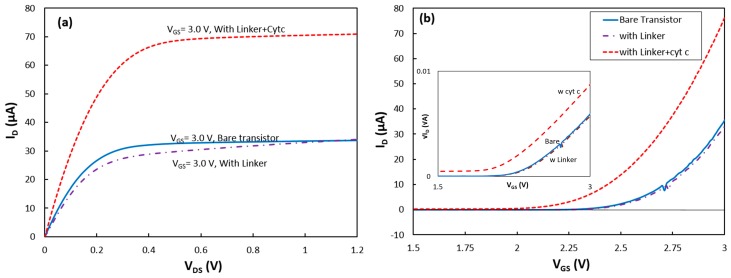
(**a**) Output and (**b**) transconductance (at *V_DS_* = 1.0 V) characteristics of the transistor in the dark for the bare device, with the SAM linker layer, and immobilized cytochrome on the gate insulator (Si_3_N_4_). (Inset) √*I_D_* − *V_GS_* at *V_DS_* = 1.0 V used for estimating *C_G_* and *V_th_*.

First, a bare transistor was tested in the dark box. The output characteristic of the device was obtained by measuring the drain current when the gate voltage (i.e., reference electrode) was kept constant and the drain voltage was scanned from 0.0 V to 1.2 V. The experiment was repeated for different gate voltages from 2.0 V to 3.0 V. The *I_D_* − *V_DS_* of the transistor when the gate voltage was at 3.0 V is shown in Figure 2a. The output characteristic shows that the device was operating in the saturation mode when *V_DS_* was 1.0 V. Therefore, the transconductance characteristic of the device was measured by recording the drain current when *V_DS_* = 1.0 V, and the gate voltage was scanned from 0.0 V to 3.0 V (Figure 2b). The output and transconductance characteristics of the device were measured at different wavelengths when the transistor was exposed to monochromatic and white light from the LEDs.

To study the success rate in immobilizing RCs via the new linker structure, first a SAM layer was deposited on the transistor. After the deposition, the sample was characterized in the absence and presence of illumination. The dark output and transconductance responses of the device with the linker coating are shown in Figure 2a,b, respectively. The slight change in the dark response of the transistor indicates formation of the SAM on the Si_3_N_4_ layer.

The device was removed from the cell for deposition of cytochrome on the SAM. After the deposition, the transistor was returned to the test cell inside the dark box, and it was found that the transistor drain current was greatly enhanced. Under a gate-source voltage (*V_GS_*) of 3 V, the saturation current at *V_DS_* = 1.2 V increased from 34 μA for the transistor without cyt *c* to 71 μA after adsorption of the protein (Figure 2a).

The drain current, *I_D_*, in a FET device in the saturation mode when *V_GS_* is greater than the threshold voltage, *V_th_*, is expressed by [16]:

(1) $$I_{D}=\frac{\mu_{Si}C_{G}}{2}\frac{W}{L}{\left(V_{GS}-V_{th}\right)}^{2},$$

where *μ_Si_* is the mobility of the carrier in the channel of the transistor, *W*/*L* is the channel width to length ratio, and *C_G_* is the gate capacitance which is independent from *W* and *L*. For the bare transistor:

(2) $$C_{G}={\left(\frac{t_{SiO2}}{\varepsilon_{SiO2}}-\frac{t_{Si3N4}}{\varepsilon_{Si3N4}}\right)}^{-1},$$

where *t*_*SiO*2_, *t*_*Si*3*N*4_, *ε*_*SiO*2_ and *ε*_*Si*3*N*4_ are the thickness and permittivity of the SiO_2_ and Si_3_N_4_ layers, respectively. Considering that the carrier’s mobility in the silicon (buried under the dielectric layers) and *W*/*L* have not been changed after coating the device with the protein, the change in the drain current should be due to a change in *C_G_* and/or *V_th_*. To study the effect of linker and cyt *c* on the device parameters, the transconductance responses were converted to √*I_D_* vs *V_GS_* (inset of Figure 2b). As shown in Equation (3), the √*I_D_* − *V_GS_* curves were approximated with linear functions for *V_GS_* > 2.75 V.

(3) $$\surd I_{D}=\surd \left(\frac{\mu_{Si}C_{G}}{2}\frac{W}{L}\right)\left(V_{GS}-V_{th}\right),$$

Considering that the carrier’s mobility in the silicon (buried under the dielectric layers) and *W*/*L* have not been changed after coating the device with the linker and protein, changes in the slope of the linear functions were interpreted as changes in the *C_G_* value, and the threshold voltage was found from the *V_GS_*-intercept of the linear functions.

The data show that *C_G_* dropped by 4% after the SAM coating, but the slope did not change after the cytochrome deposition. Due to the thick layers of SiO_2_ and Si_3_N_4_ and thin insulating SAM, this change in *C_G_* is reasonable. However, a relatively large reduction of the threshold voltage from 2.39 V for the bare transistor to 2.17 V after the cytochrome coating on the top of the SAM was observed. The threshold voltage in a fabricated transistor can be changed if charged molecules attach to the dielectric layer [9]. The density of charges, *Q_s_*, from the attached molecules can be estimated from the change in the threshold voltage, Δ*V_th_*, using the simple equation of *Q_s_* = Δ*V_th_* × *C_G_* [16]. Knowing the thickness and the permittivity of the SiO_2_ and Si_3_N_4_ layers, the *C_G_* value was estimated for the bare transistor to be 2.30 × 10^−8^ F/cm^2^. Therefore, *Q_s_* was found to be 5.12 × 10^−9^ C/cm^2^. Assuming that the charge on the surface of the protein is passivated by the counter ions in the electrolyte, the effective charge of the reduced cyt *c* would be only two unit charges (unit charge = 1.6 × 10^−19^ C) from the unshielded charge at the heme (Fe^2+^) core of the protein. This corresponds to the surface density of attached cyt *c*s to be 1.6 × 10^10^ proteins per cm^2^. Considering the protein dimensions, this implies only ~15% of the surface coverage. Although surface coverage near 100% is achievable on a gold electrode (with a different linker molecule) [17], immobilizing cyt *c* to Si_3_N_4_ has not been reported before. In this work, carboxydecylphosphonic acid which is recommended as a linker for various insulating materials was chosen. However, a separate study would be required to find the optimum deposition conditions to achieve a compact linker layer on Si_3_N_4_. Therefore, it is likely that the linker coverage was limited which resulted in the poor surface coverage by the proteins. The transconductance characteristic of the device was also measured under other illumination conditions, and the results were consistent with the above interpretations.

**Figure 3:**
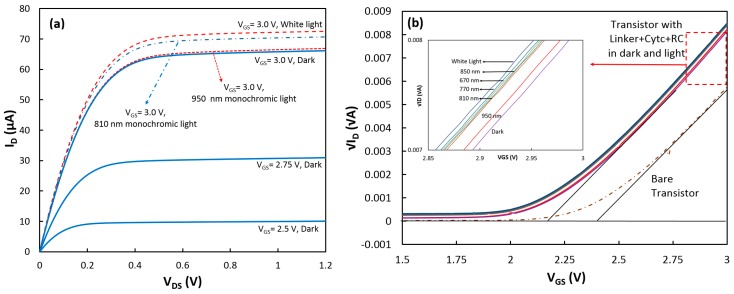
(**a**) Output and (**b**) transconductance (at *V_DS_* = 1.0 V) characteristics of the transistor with the linker+cyt c+RC on top of the gate under dark and light conditions. (Inset in (**b**)) The linear approximation to √*I_D_* − *V_GS_* for the device in the dark and under illumination of white and monochromatic lights.

After the measurements, the device was removed from the test cell for the RC incubation. The transistor with the linker, cytochrome c, and RC (linker+cyt *c*+RC) was tested again in the dark and illuminated conditions in 0.1 M Tris-HCl buffer (pH 8.0) electrolyte containing 0.05 M methyl viologen. Figure 3a shows the output characteristic of the device for three different gate voltages when it was in the dark, and the *I_D_* − *V_DS_* curves for *V_GS_* = 3.0 V under white and two monochromatic lights. The largest change in *I_D_* was measured when white light was applied to the device. The change in the transistor drain current under illumination suggests that the charge density at the gate of the transistor was increased (reduction of threshold voltage). Since it is expected that RCs would be attached from the P-side to cyt *c*, one explanation is that P^+^ in the illuminated RCs would receive an electron from cyt *c* (oxidizing cyt *c*, Fe^3+^) while the negative charge would be transferred to MV and diffused to the bulk electrolyte. Therefore, only RCs that were coupled to cyt *c*s would enhance *Q_s_*. It should be mentioned that due to the insulating properties of the gate insulator, there is no charge transfer between cytochrome and the transistor channel. In fact, the advantage of this method is that the threshold voltage changes only with the charge density, not the charge transfer rate (photocurrent). The charge density is directly proportional to the number of the attached proteins.

**Figure 4:**
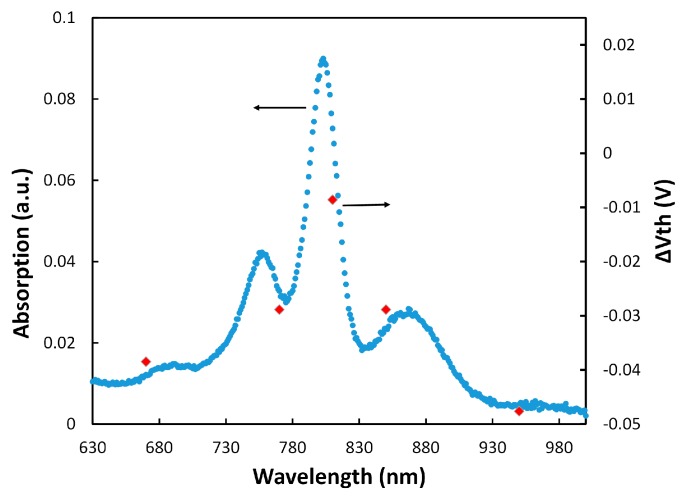
The absorption spectrum of the RC and the results from Δ*V_th_* under the tested wavelengths.

Similar to the approach with cytochrome, the additional charge density generated by the coupled RCs under illumination was estimated to be 8.34 × 10^−10^ C/cm^2^ from the threshold voltage shift in the transistor (Figure 3b). Considering that each RC can add one positive charge, the density of the properly attached RCs to cyt *c*s was estimated to be 5.21 × 10^9^ (= 8.34 × 10^−10^/1.6 × 10^−19^) proteins/cm^2^. This implies coupling success rate of 33% (5.21 × 10^9^/1.6 × 10^10^) for RCs to cyt *cs* which means that for every three cytochrome proteins on the SAM layer, one can successfully couple to RCs. To confirm that the generated charges were actually from RCs, Δ*V_th_* (*V_th w RC_* − *V_th wo RC_*) was calculated at various wavelengths when the transistor was illuminated with monochromatic LEDs with low intensity. Figure 4 shows that Δ*V_th_* follows the absorption spectrum of RCs.

The results in Figure 4 also show that the electrochemical transistor with immobilized RCs could be used as a photosensor for detecting low light intensities. It should be mentioned that we have tested different RC immobilization configurations for making a photosensor, including only the RC, linker+RC, and cyt *c*+RC on the Si_3_N_4_ gate insulator. Although some shift in the threshold voltage was observed in those configurations, the monochromatic illumination tests did not show any pattern (like the one in Figure 4) to verify generation of the gate charges by the RC. This confirmed that the linker+cyt *c*+RC structure generates an RC orientation for effective coupling to the cyt *c* protein.

## 4. Conclusions

A sequential deposition approach of a linker molecule, cyt *c* and RC proteins was implemented on the gate insulator of an electrochemical transistor to study the concentration of cytochrome and coupled RCs. It was found that the ratio of coupled RC to cytochrome can be as high as 1 to 3. The combination of the immobilized RCs to the gate of the transistor also showed the potential in application of a new bio-photosensor for detecting low intensity lights.
